# *Fasciola hepatica* GST downregulates NF-κB pathway effectors and inflammatory cytokines while promoting survival in a mouse septic shock model

**DOI:** 10.1038/s41598-018-37652-x

**Published:** 2019-02-19

**Authors:** Vasti Aguayo, Bianca N. Valdés Fernandez, Madeline Rodríguez-Valentín, Caleb Ruiz-Jiménez, Marcos J. Ramos-Benítez, Loyda B. Méndez, Ana M. Espino

**Affiliations:** 1 University of Puerto Rico, Medical Sciences Campus, Department of Microbiology, San Juan, Puerto Rico; 2 Department of Microbiology, Universidad Central del Caribe, Bayamon, Puerto Rico; 3 University of Puerto Rico, Rio Piedras Campus, Department of Biology, San Juan, Puerto Rico; 4 School of Science & Technology Universidad del Este, Carolina, Puerto Rico

## Abstract

Parasitic helminths and helminth-derived molecules have demonstrated to possess powerful anti-inflammatory properties and confirmed therapeutic effects on inflammatory diseases. The helminth *Fasciola hepatica* has been reported to suppress specific Th1 specific immune responses induced by concurrent bacterial infections, thus demonstrating its anti-inflammatory ability *in vivo*. In this study, we demonstrate that native *F*. *hepatica* glutathione S-transferase (nFhGST), a major parasite excretory-secretory antigen, majorly comprised of Mu-class GST isoforms, significantly suppresses the LPS-induced TNFα and IL1β of mouse bone-marrow derived macrophages *in vitro* and the pro-inflammatory cytokine/chemokine storm within C57BL/6 mice exposed to lethal doses of LPS increasing their survival rate by more than 85%. Using THP1-Blue CD14 cells, a human monocyte cell line, we also demonstrate that nFhGST suppresses NF-κB activation in response to multiple TLR-ligands, including whole bacteria clinical isolates and this suppression was found to be dose-dependent and independent of the timing of exposure. Moreover, the suppressive effect of nFhGST on NF-κB activation was shown to be independent of enzyme activity or secondary structure of protein. As part of its anti-inflammatory effect nFhGST target multiple proteins of the canonic and non-canonic NF-κB signaling pathway as well as also JAK/STAT pathway. Overall, our results demonstrate the potent anti-inflammatory properties of nFhGST and its therapeutic potential as an anti-inflammatory agent.

## Introduction

Parasitic helminths have co-evolved with their hosts for countless years and developed multiple and sophisticated mechanisms to modulate the host’s immune system to ensure their survival^1^. As part of their immunomodulatory mechanisms, all helminths establish a regulatory anti-inflammatory Th2 immune response in their mammalian host^1,2^, which is thought to be mutually beneficial for host and parasite, because it protects the host from severe consequences of inflammatory responses, while preventing the elimination of worms^3^. Thus, a large number of human and animal studies have demonstrated that helminth infections could be used to ameliorate or prevent autoimmune diseases and allergies^4–7^. These studies have helped incorporate helminth infections into the expanded ‘Hygiene Hypothesis’. *Fasciola hepatica*, one of the most prevalent parasitic Platyhelminths, is not an exception.

*F*. *hepatica* causes fascioliasis, an emergent neglected tropical disease that affects around 17 million persons worldwide and also infects livestock, causing economic losses estimated around $3 billion annually^8^. Throughout the earliest phases of infection the invading parasite secretes a myriad of molecules, termed excretory-secretory products (ESPs) that are responsible for inducing Th2-immune responses simultaneously with the suppression of Th1cytokines^9,10^. Glutathione S-transferases (GSTs), major components of *F*. *hepatica* ESPs, are a family of multifunctional enzymes that constitute approximately 4% of the total soluble parasite protein^11^. The GST-super family is essential for the parasite survival due to its detoxification and xenobiotic clearance functions^12^. The GST family is comprised of at least seven isoforms, of which five have already been cloned. The cloned isoforms Fh51, Fh47, Fh7, and Fh1 belong to the Mu-class GSTs and share a sequence identity higher than 71%^13^. The fifth cloned isoform belongs to the Sigma class^11,14^ and shares a sequence identity of ~25% with other GST-classes^14^. A previous study demonstrated that a recombinant variant of *F*. *hepatica* GST-Sigma induces a partial activation of dendritic cells (DCs), activates mitogen-activated protein kinases (MAPKs) and the nuclear factor-κB (NF-κB), all of which are dependent on its enzymatic activity^15^. Interestingly, this same group initially tested recombinant *F*. *hepatica* GST-Mu, and reported that it did not induce cytokine secretion^15^, suggesting that DC modulation could be subject to subclass specificity and enzyme activity.

In this study, we purified native forms of *F*. *hepatica* GSTs containing Mu-class isoforms as major components (named nFhGST) from adult fluke extract and investigated the anti-inflammatory properties of the purified protein *in vivo* and *in vitro*. To our knowledge, this study is the first to report the potent anti-inflammatory properties of *F*. *hepatica* GST-Mu isoforms, providing evidence of its broad spectrum of action.

## Results

### Native *F*. *hepatica* GST purification, integrity and composition

GSTs are enzymes with high affinity towards reduced glutathione (GSH). Our group and others have applied a method based on GSH-affinity chromatography as part of the strategy to identify GST isoforms from a cytosolic extract of *F*. *hepatica*^14,16^. In the present study, the protein was purified in active form with an average specific enzymatic activity of ~35 Units/min/mg (two separate batches) and when analyzed by 12.5% SDS-PAGE, it showed a single homogenous polypeptide band of ~25 kDa with a high purity level (96.17%) as demonstrated by the iBAQ value from the nano-LC MS/MS analysis^17^. The analysis also revealed that the purified protein was comprised of five isoforms belonging to the Mu-class and one Sigma-class isoform (Table S1, Fig. S1). Thus, for the purpose of this work the purified protein was termed nFhGST.

### nFhGST suppressed the expression of IL1β and TNFα induced by LPS in murine macrophages *in vitro*, suppressed the inflammatory responses and protected mice from endotoxemia

**Figure 1:**
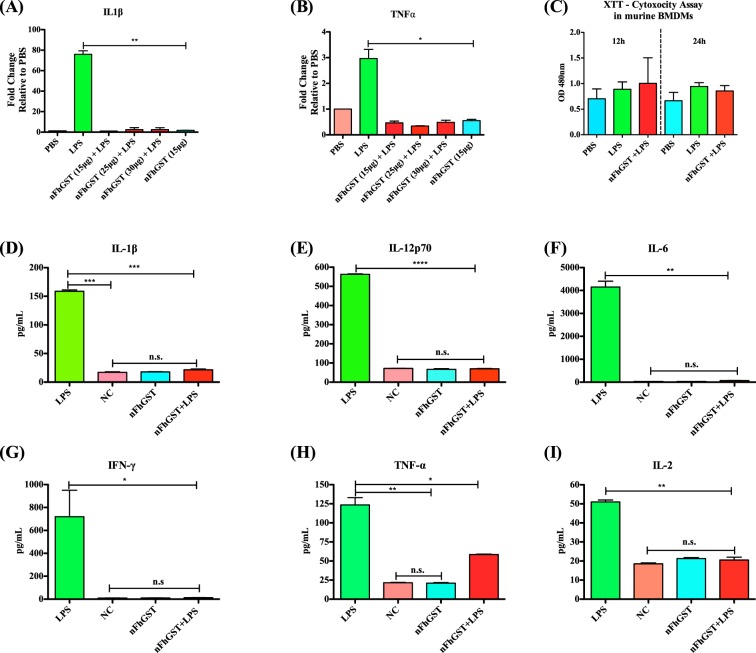
nFhGST suppresses the expression of IL-1β and TNFα from murine macrophages stimulated with LPS *in vitro* and prevent the development of the cytokine storm in a murine model of septic shock. Bone marrow derived macrophages (BMDM) from naïve C57BL6 mice were treated with 10 μg/ml nFhGST 30 min before stimulation with LPS (100 ng/ml). Control cells were treated with LPS or PBS alone. After treatments cells were incubated by 18 h, at 37 °C 5% CO_2_. Expression of IL-1β and TNFα was measured by real-time RT-PCR. Results shown are expressed as fold-changes in expression relative to cells stimulated with PBS and presented as mean ± SD of a minimum of three experiments, each in triplicate. nFhGST significantly suppressed the expression of IL-1β (**A**) and TNFα (**B**) (**p = 0.0021 and *p = 0.021), respectively. (**C**) This effect was not caused by toxicity. C57BL/6 mice, 6 to 8 weeks old (n = 12 per group) received a single intraperitoneal (i.p) injection with nFhGST (200 μg for each mouse), 1 h before i.p. injection with 1 mg LPS (*E*. *coli* 0111:B4, 60 mg/kg of weight per mouse). Control mice received PBS-endotoxin free, nFhGST or LPS only (i.p). Mice were monitored by mortality for 48 h. Animals that were about to die during experiment, or that survived the experiment, were sacrificed by cervical dislocation and blood samples were collected from the orbital vein or cardiac puncture. Concentrations of serum (**D**) IL1β (***p = 0.003), (**E**) IL12p70 (****p < 0.0001), (**F**) IL-6 (**p = 0.0038), (**G**) INFγ (*p = 0.001), (**H**) TNFα (**p = 0.0086) and (**E**) IL-2 (**p = 0.0012) were measured by a mouse cytokine/chemokine assay.

Since *F*. *hepatica* antigens, such as fatty acid binding proteins or tegument have shown to suppress the expression of pro-inflammatory cytokines in murine bone marrow derived macrophages^18^ and specific Th1-type immune responses induced by bacterial infections or their endotoxin^19,20^, we proceeded to determine whether nFhGST could have a similar function. We extracted cells from mouse bone marrow, differentiated them into macrophages *in vitro* (BMDMs) and then exposed cells to different nFhGST concentrations ranging 15 to 30 μg/ml prior to stimulation with LPS. The expression of tumor necrosis factor-α (TNFα) and interleukin-1β (IL-1β) was measured by real-time RT-PCR 18 h after LPS-stimulation. As expected, BMDMs stimulated with LPS overexpressed high levels of TNFα and IL-1β, which are a signature of an ongoing inflammatory response^21,22^. In contrast, nFhGST stimulated cells failed to express TNFα and IL-1β cytokines, when they were exposed to three different concentrations of nFhGST in combination with LPS. The minimal nFhGST concentration tested (15 μg/ml) rendered maximal significant suppression (*p* = 0.021 and *p* = 0.0021, respectively) for both cytokines (Fig. 1A,B). To rule out that the suppressive effect showed by nFhGST on BMDM is due to toxicity, we measured the influence of nFhGST treatment on cell viability using an XTT assay after 12 h and 24 h of treatment. Results demonstrated that nFhGST alone or combined with LPS did not compromise the cell viability (Fig. 1C).

**Figure 2:**
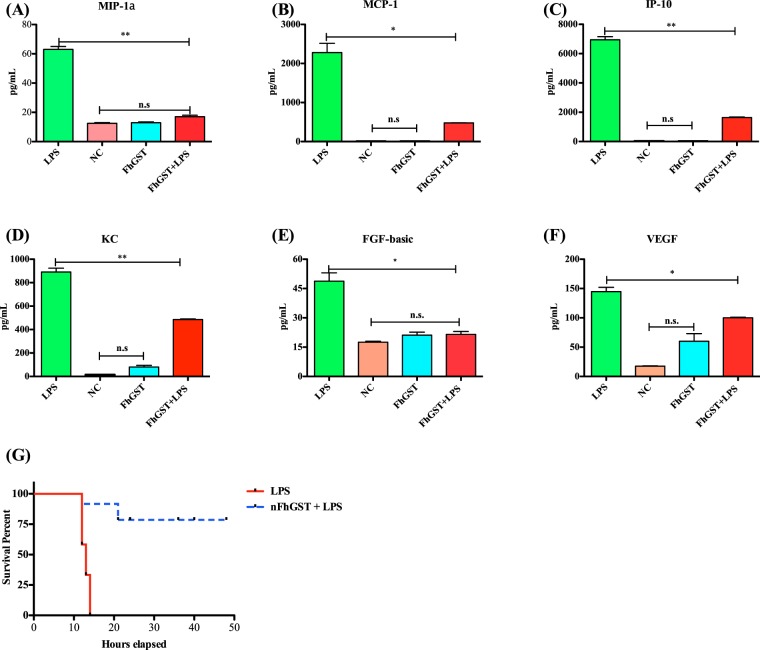
nFhGST suppressed pro-inflammatory chemokines and protected mice from endotoxin shock. Blood samples collected from animals that were about to die or survived the LPS-lethal challenge injection as described in figure-1, were tested for determining chemokine levels, which were measured by a cytokine/chemokine mouse cytokine array. **(A)** MIP-1a (****p < 0.0001), **(B)** MCP-1 (*p = 0.0166), **(C)** IP-10 (**p = 0.0017), **(D)** KC (**p = 0.0014), **(E)** FGF-basic (*p = 0.0182) and **(F)** VEGF (*0.0123). **(H)** Animals were monitored for mortality for 48 h after the LPS-challenge.

Next, we proceeded to determine whether nFhGST could be able to suppress the production of pro-inflammatory cytokines *in vivo* and for this we developed a mouse model of septic shock using C57BL/6 mice, which is the prototypical Th1-type mouse strain^23^. As expected, a single intraperitoneal injection with a lethal dose of 60 mg/kg LPS^24^ induced significantly higher levels of serum IL-1β (*p* = 0.0003), IL-12p70 (*p* < 0.0001), IL-6 (*p* = 0.0038), TNFα (*p* = 0.0086), IL-2 (*p* = 0.0012), IFNγ (*p* = 0.09), MIP-1α (*p* = 0.0017), MCP-1 (*p* = 0.0106), KC (*p* = 0.0014), FGF-basic (*p* = 0.0182) and VEGF (*p* = 0.0032) compared with the injection of PBS or nFhGST. However, 200 μg nFhGST, a concentration arbitrarily selected based on studies with unrelated molecules^24^, applied 30 min before LPS injection resulted in significantly reduced levels of IL-1β (p = 0.0004), IL-12p70 (p < 0.0001), IL-6 (p = 0.0039), TNFα (p = 0.0208), IL-2 (p = 0.0035) and IFNγ (p = 0.01) cytokines (Fig. 1D–I) as well as significantly reduced levels of MIP-1α (p = 0.0024), MCP-1 (p = 0.0166), KC (p = 0.0067), FGF-basic (p = 0.0263) and VEGF (p = 0.0257) compared with LPS alone (Fig. 2A–F). Moreover, nFhGST increased the survival rate in LPS-challenged animals by 85% (Fig. 2G). Furthermore, these results were replicated in BALB/c mice, the prototypical Th2-type mouse strain^23^ injected with a lethal doses of 7 mg/kg body weight^25^ (Figs S2 and S3), suggesting that nFhGST could be used as potential anti-inflammatory agent to block the adverse biological consequence of septic shock *in vivo* regardless of the genetic background of mice.

### nFhGST suppressed the LPS-induced NF-κB activation in THP1-Blue CD14 cells independently of its secondary/tertiary structure integrity

**Figure 3:**
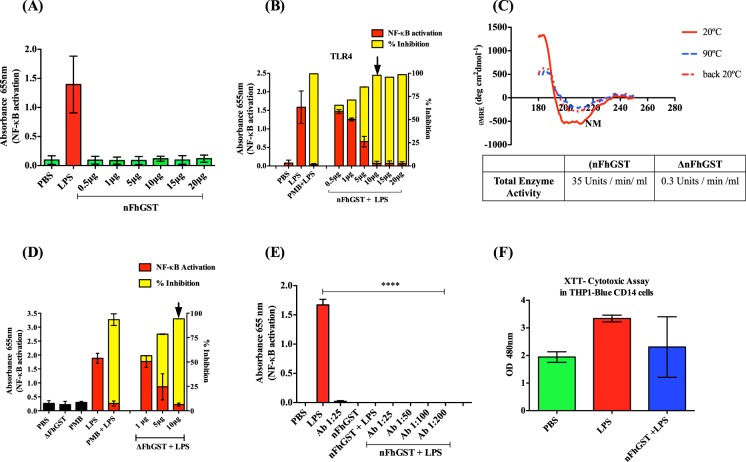
nFhGST does not activate NF-κB but blocks the LPS-induced activation and this effect is not modified after a heat-protein denaturation process or in the presence of anti-FhGST antibodies. THP1 Blue CD14 cells were seeded at 1 × 10^6^ cells/well in 96 well flat-bottom plates. The levels of SEAP secreted to the culture media as indicator of NF-κB activation were estimated by reading at 655 nm, 18 h after treatment with FhGST and/or LPS-stimulation. The inhibition percentage in the levels of SEAP secreted to culture media were calculated by the formula R (%) = 1 − [(A–C)/(B,C)] × 100, where A is the mean A_655_ of three replicates obtained when cells were cultured with nFhGST, and B is the mean A_655_ value obtained when cells were exposed to TLR-ligands, and C is the mean A_655_ of three replicates obtained when cells were stimulated with PBS. (**A**) Cells were treated with nFhGST at different concentrations (0.5 to 20 μg/ml). (**B**) Cells were treated with different concentrations of nFhGST for 30 min and then stimulated with LPS (1 μg/ml). (**C**) Circular Dichroism spectra of nFhGST at 20 °C, 90 °C (coarse dashed line) and returning 20 °C (thin dashed line). Spectra were obtained using the protein at 0.1 mg/ml. Enzyme activity of nFhGST before and after denaturing (ΔFhGST) was measured by CDNB enzymatic activity assay. (**D**) Cells were first treated with different concentrations of denatured protein (ΔFhGST) 30 min before LPS (1 μg/ml) stimulation. (**E**) Cells were exposed first treated for 30 min with an anti-nFhGST antibody of high titer at different dilutions (1:25 to 1:200), next were cultured with nFhGST (10 μg/ml) and 30 min later were stimulated with LPS (1 μg/ml). (F) Cell viability was determined by adding 50 μl XTT to each well. The absorbance of each well was read at 480 nm.

Given the potent anti-inflammatory effect of nFhGST on murine macrophages *in vitro* and endotoxemia *in vivo*, we hypothesized that nFhGST could be exerting an antagonist effect on TLR-4-signaling consequently altering LPS-induced NF-κB activation and suppressing the production of pro-inflammatory cytokines. To address this hypothesis, we performed *in vitro* experiments using the reporter cell line THP1-Blue-CD14, which is a cell line derived from human monocytes that express multiple TLRs and that has been stably transfected with a reporter plasmid expressing a secreted embryonic alkaline phosphatase (SEAP) gene under the control of a promoter inducible by the nuclear transcription factor-κB (NF-κB). In this system, upon TLR stimulation, activated cells secrete SEAP into the culture media indicating that NF-κB has been activated and translocated into the nucleus. The levels of SEAP are easily measurable at 655 nm using QUANTI-Blue^TM^. First, we treated cells with increasing concentrations of nFhGST alone or in the presence of LPS (1 μg/ml). Results show that nFhGST was unable to promote NF-κB activation at any of concentrations tested (Fig. 3A). However, it was able to block the LPS-induced NF-κB activation in a dose-dependent manner. Thus, the lowest nFhGST concentrations tested (0.5 and 1 μg/ml), were able to suppress the LPS-induced NF-κB levels by 7% ± 0.053 and 21.0% ± 0.037, respectively. Suppressions increased with nFhGST concentration and reached maximal values (≥95.0% ± 0.055) at nFhGST concentration ≥10 μg/ml (Fig. 3B).

Enzymatic activity is often used to distinguish between seven independent classes of known cytosolic GSTs based on inhibitor sensitivity and substrate specificity^26^. To determine whether the enzymatic activity is required for the capacity of nFhGST to suppress LPS-induced NF-κB activation in THP1-Blue CD14 cells experiments similar to the ones described previously were conducted with a heat-denatured protein. nFhGST is a protein with a high content of α–helical regions in its protein moiety as demonstrated by the positive absorption at ~193 nm and the negative absorption between 200 nm and 220 nm determined by circular dichroism (CD)-spectra analysis at 20 °C^27^. When nFhGST was submitted to denaturing temperature of 90 °C, this absorption spectra became distorted indicating the loss of the secondary structure of the molecule, which never returned to its native form even when the temperature returned to 20 °C. Furthermore, the heat treatment also produced irreversible loss in the enzymatic activity of nFhGST **(**Fig. 3C). When cells were treated with different concentrations of denatured nFhGST (ΔFhGST) and then stimulated with LPS, the NF-κB activation was also dose-dependent suppressed reaching maximal suppression values of ~88% at ≥10 μg (Fig. 3D). This indicates that the capacity of nFhGST to block the LPS-stimulation of TLR4 is independent of its enzymatic activity, as well as its native secondary/tertiary structure. Additionally, because nFhGST is an immunogenic molecule capable of inducing antibodies during the active infection^16^, we wanted to ascertain whether the presence of high levels of anti-nFhGST antibodies could impact the antagonistic capacity of this molecule on TLR4. To answer this question, THP1-Blue CD14 cells were incubated with different dilutions of an anti-nFhGST serum followed by the addition of nFhGST and the stimulation with LPS. Results showed that even at the highest serum concentration (1:25 dilution), the antibody did not affect nFhGST’s capacity of suppressing the LPS-induced NF-κB activation (Fig. 3E). Lastly, we measured the viability of cells and demonstrated that the observed results were not attributed to cell toxicity (Fig. 3F).

### nFhGST targets multiple Toll-like receptors (TLRs) in a human monocyte cell line and this effect is independently of its secondary/tertiary structure integrity

**Figure 4:**
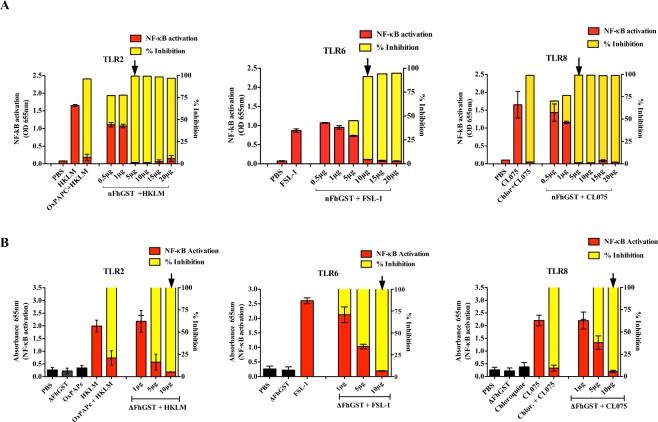
nFhGST suppressed the NF-κB activation induced by multiple TLRs in a manner that is dose-dependent and independent of TLR-ligand exposure. THP1 Blue CD14 cells were seeded at 1 × 10^6^ cells/well in 96 well flat-bottom plates, treated with (**A**) nFhGST or (**B**) denatured FhGST (ΔFhGST) (different concentrations) and then stimulated with HKLM (10^8^ cells/ml), FSL-1 (1 μg/ml) and CL075 (10 μg/ml). After 18 h, the QB-reagent was added to culture and readings at 655 nm were done 4 h later. The levels of SEAP secreted to the culture media as indicator of NF-κB activation were estimated by reading at 655 nm 18 h after treatment with FhGST and/or TLR-ligands stimulation. The inhibition percentage in the levels of SEAP secreted to culture media were calculated by the formula R (%) = 1−[(A–C)/(B-C)] × 100, where A is the mean A_655_ of three replicates obtained when cells were cultured with nFhGST, B is the mean A_655_ value obtained when cells were exposed to TLR-ligands and C is the mean A_655_ of three replicates obtained when cells were stimulated with PBS. Arrow indicates the nFhGST concentration that renders maximal NF-κB suppression.

**Figure 5:**
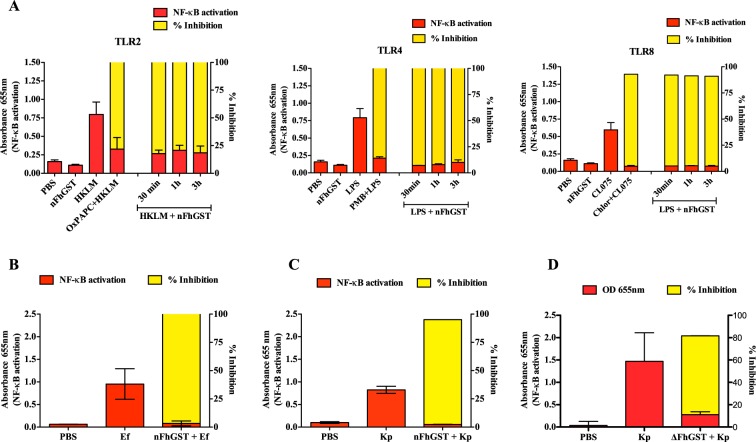
nFhGST suppresses the NF-kB activation induced by multiple TLR-ligands after the onset and in response to whole bacteria. THP1 Blue CD14 cells were seeded at 1 × 10^6^ cells/well in 96 well flat-bottom plates. **(A)** Cells were first stimulated with HKLM (10^8^ cells/ml), LPS (1 μg/ml), CL075 (10 μg/ml) for 30 min, 1 h and 3 h and treated with nFhGST (10 μg/ml). **(B)** Cells were treated with nFhGST (10 μg/ml) and then simulated with *Enterococcus faecalis* (1.14 × 10^9^ cells/ml) or **(C)** *Klebsiella pneumoniae* (1.22 × 10^8^ cells/ml). (**D**) Cells were first treated with denatured FhGST (ΔFhGST) (10 μg/ml) and then stimulated with whole *Klebsiella pneumonia*. After 18 h of the QB-reagent was added to culture and readings at 655 nm were done 4 h later. The levels of SEAP secreted to the culture media as indicator of NF-κB activation were estimated by reading at 655 nm 18 h after treatment with FhGST and/or TLR-ligands or whole bacteria stimulation. The inhibition percentages in the levels of SEAP secreted to culture media were calculated as described in figure-4.

To ascertain whether nFhGST could counteract the NF-κB activation triggered by TLRs-ligands other than LPS, THP1-Blue-CD14 cells were exposed to increasing concentrations of nFhGST in the presence and absence of heat-killed *Listeria monocytogenes* (HKLM, 10^8^ cells/ml), synthetic diacylated lipoprotein (FSL-1, 1 μg/ml) and orthiazoloquinoline (CL075, 10 μg/ml), which are specific ligands for TLR2, TLR6 and TLR8, respectively. Each of these specific ligands alone induced maximal NF-κB -activation. However, in the presence of nFhGST, the activations produced by these ligands were sequentially suppressed with increasing concentrations of nFhGST, reaching maximal reductions (>90%) at concentrations of ≥ 5 or  10 μg/ml (Fig. 4A). Similar behavior was observed when THP1-Blue CD14 cells were treated with the denatured protein (ΔFhGST) (Fig. 4B). Moreover, we also determined that nFhGST significantly suppressed the NF-κB activation induced by all TLR-ligands studied, including LPS even when nFhGST was added to culture up to 3 h after stimulation (Fig. 5A). Collectively, these results suggest that nFhGST possesses dose-dependent/time-independent anti-inflammatory capabilities via multiple TLRs.

In an attempt to mimic an *in vitro* setting of bacterial infection, THP1-Blue-CD14 cells treated with nFhGST were stimulated with optimized concentrations of two heat-attenuated clinical bacterial isolates: *Klebsiella pneumoniae* (1.22 × 10^8^ cells/ml) and *Enterococcus faecalis* (1.14 × 10^9^ cells/ml), which are representative of Gram-negative and Gram-positive bacterial strains, respectively. As expected, nFhGST significantly reduced the NF-κB activation induced by these bacteria strains by 92.7% (*p* < 0.0001) and 91.4% (*p* < 0.0001), respectively (Fig. 5B,C), which validates the results obtained with the separates TLR’s agonists. When denatured FhGST was tested, it also reduced the NF-κB activation induced by *K*. *pneumoniae* by 81.6% (*p* < 0.001) (Fig. 5D).

### nFhGST does not bind to the polysaccharide core of LPS-endotoxin

**Figure 6:**
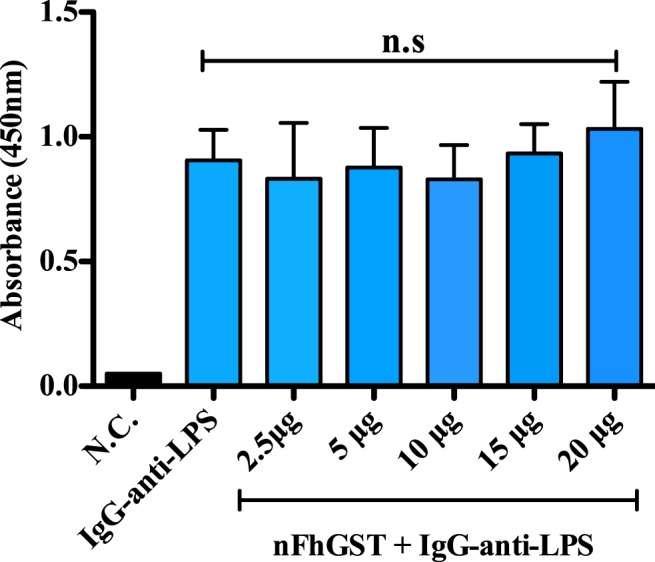
nFhGST does not bind to LPS. A modified EndoCab^R^ IgG assay (HK504-IgG, Hycult Biotech) was used. nFhGST concentrations (2.5 μg to 20 μg) were added in duplicate to microtiter wells coated with LPS from four Gram negative bacterial species. After 1 h incubation at 37 °C, the IgG antibody was added (100 μl/well) (diluted 1:8) followed by the sequential addition of peroxidase conjugate antibody and TMB substrate solution. After 30 min of incubation at room temperature in the dark, the reaction was stopped and absorbance at 450 nm was read. Wells incubated with the IgG standards in absence of nFhGST were used as positive controls. Wells incubated only with nFhGST in absence of standards, or wells incubated with wash buffer in absence of nFhGST and standards, were used as negative controls.

Given nFhGST was able to block the activation of TLR4 induced by LPS endotoxin of two different bacterial sources (*E*. *coli* and whole *K*. *pneumoniae*), we wanted to investigate whether it could be binding to LPS. For this purpose, we used an EndoCab IgG assay (Hycult Biotech, Cat No. HK504-IGG) that was customized into an inhibition anti-endotoxin core antibody assay. nFhGST, at different concentrations (2.5 to 20 μg), was incubated in duplicate on microtiter wells coated with equimolar amounts of endotoxin rough-LPS from four Gram-negative bacterial species, each comprised of a complete inner core, but lacking the O-specific polysaccharide chain. After 1 h incubation at 37 °C and the corresponding wash steps, the specific IgG against the endotoxin core was added to wells and incubated for 1 h, which was followed by the peroxidase conjugated antibody and the substrate addition. Results demonstrated that nFhGST was unable to impede the binding between the specific IgG standards and the endotoxin core coated on the wells at any of concentrations tested (Fig. 6), which demonstrated that nFhGST does not bind to the LPS endotoxin.

### nFhGST suppressed multiple proteins from the NF-κB signaling pathway

**Figure 7:**
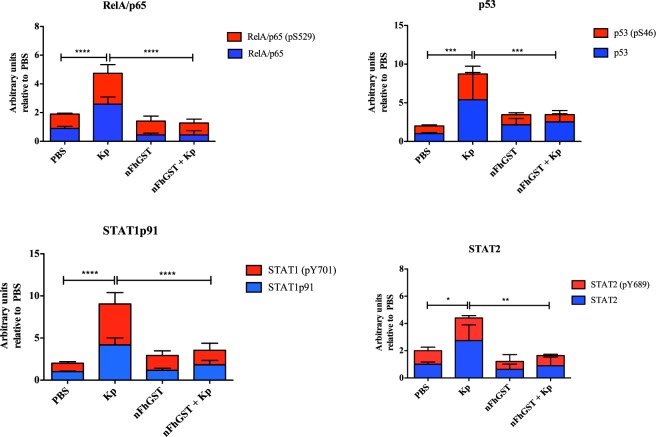
nFhGST suppressed the levels of four phosphorylated proteins that participate in the NF-κB -pathway. THP1 Blue CD14 cells seeded at 1 × 10^6^ cells/well in 96 well flat-bottom plates were cultured with nFhGST (10 μg/ml) 30 min before stimulation with whole bacteria extract of *K*. *pneumoniae* (1.22 × 10^8^ cells/ml). The levels of SEAP secreted to the culture media as indicator of NF-κB activation were estimated by reading at 655 nm, 18 h after TLR-ligand stimulation (Reduction >85% in NF-κB activation). Cells treated with PBS, *K*. *pneumoniae* or nFhGST alone were used as controls. Cell lysates were diluted and incubated overnight with the Human NF-κB pathway array. Dot blot images were acquired in a Chemidoc MP Imaging system (Bio Rad, Hercules, CA) and densitometry analysis was performed on all the arrays using ImageJ software. Values were expressed as arbitrary units over the PBS-treated cells. nFhGST significantly suppressed total and phosphorylated RelA/p65 (****p < 0.0001), phosphorylated p53 (***p < 0.0001), total and phosphorylated STAT1 (****p < 0.0001) and total and phosphorylated STAT2 induced by *K*. *pneumoniae* (*p < 0.011, **p = 0.005).

**Figure 8:**
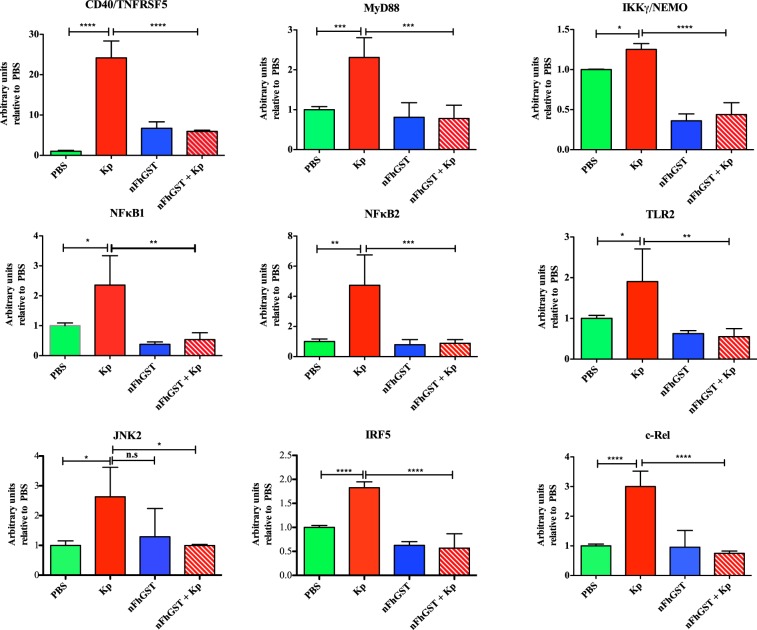
nFhGST suppressed the levels of multiple proteins participating in the canonic and non-canonic NF-κB –pathway as well as in the JAK/STAT pathway. THP1 Blue CD14 cells seeded at 1 × 10^6^ cells/well in 96 well flat-bottom plates were culture with nFhGST (10 μg/ml) 30 min before stimulation with whole bacteria extract of *K*. *pneumoniae* (1.22 × 10^8^ cells/ml). The levels of SEAP secreted to the culture media as indicator of NF-κB activation were estimated by reading at 655 nm, 18 h after TLR-ligand stimulation (Reduction >85% in NF-κB activation). Cells treated with PBS, *K*. *pneumoniae* or nFhGST alone were used as controls. Cell lysates were diluted and incubated overnight with the Human NF-κB pathway array. Dot blot images were acquired in a Chemidoc MP Imaging system (Bio Rad, Hercules, CA) and densitometry analysis was performed on all the arrays using ImageJ software. Values were expressed as arbitrary units over the PBS-treated cells. nFhGST significantly suppressed CD40 (****p < 0.0001), MyD88 (***p < 0.0002), IKKγ/NEMO (****p < 0.0001), NF-κB1 (**p = 0.0013), NF-κB2 (***p = 0.0009), TLR2 (**p = 0.003), JNK2 (*p = 0.025), IRF5 (****p < 0.0001) and cRel (****p < 0.0001).

**Figure 9:**
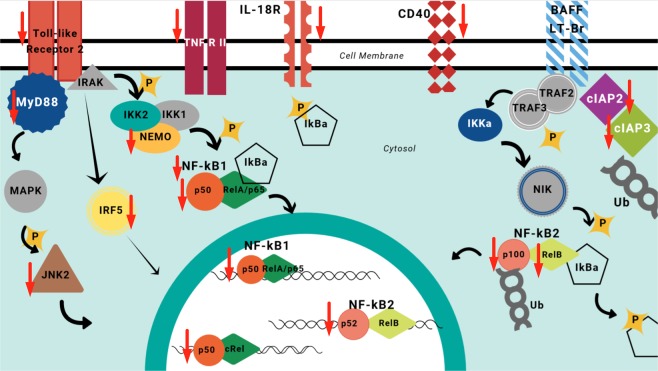
Schematic representation of canonical and non-canonical NF-κB pathway. Proteins of these pathways are up regulated once the TLRs on cells are stimulated in the presence of *Klebsiella pneumoniae*. The TLRs-activation culminates with the translocation of NF-κB into the nucleus followed for the pro-inflammatory cytokines genes. In the presence of nFhGST a large number of proteins participating in these signaling cascades are down regulated, which blocks the translocation of NF-κB into the nucleus and the cytokine synthesis. Red arrows indicate proteins that were downregulated by nFhGST.

Given the suppressive effect of nFhGST on the activation of NF-κB in response to whole bacteria, we proceeded to perform a proteome profiler array to identify the proteins of the NF-κB pathway that are suppressed by nFhGST (Fig. S4). In this experiment, we used cells stimulated with *K*. *pneumoniae* as positive control of NF-κB activation pathway via multiple TLR-engagement. The results obtained demonstrated that nFhGST was able to suppress 22 out of 27 (81.5%) proteins that were upregulated by whole heat-killed *K*. *pneumoniae*, including four phosphorylated proteins: RelAp65-pS529 (canonic NF-κB pathway), p53-pS46 (canonic pro-apoptotic molecule), STAT1-pY701 and STAT2-pY689 (JAK-STAT pathway). nFhGST also suppressed members of the NF-κB protein family (RelAp65 and cRel), including the large precursors NF-κB1 (p105) and NF-κB2 (p100); the adapter molecules MyD88, IKKγ/NEMO, the cluster differentiation-40 (CD40); and TLR2 among others. Treatment of cells with nFhGST alone did not produce any noticeable effect on the analyzed proteins based on the low-intensity signals of each spot, which were similar to the background signals observed with the PBS-stimulation (Figs 7–9, Table 1).

**Table 1:** Additional proteins of the NF-κB signaling pathway proteome array that were upregulated in THP1-Blue CD14 cells stimulated with whole bacteria extracts of *K*. *pneumonia* (Kp) and either, down regulated, or remained unchanged in the presence of nFhGST.

Protein	Arbitrary Units Relative to PBS					
	Kp	*P* value	nFhGST	*P* value	nFhGST + Kp	*P* value
BCL10	1.89 ± 0.31	*0.0478	0.79 ± 0.31	n.s	1.17 ± 0.31	n.s
cIAP1/BIRC2	1.69 ± 0.14	**0.0024	0.82 ± 0.14	n.s.	0.55 ± 0.14	**** < 0.0001
cIAP2/BIRC3	3.16 ± 0.48	**0.0036	1.65 ± 0.48	n.s.	1.35 ± 0.48	*0.0126
IL18Ra	8.65 ± 1.77	**0.0047	4.22 ± 1.77	n.s.	2.65 ± 1.77	*0.0240
IRF8	0.54 ± 0.14	*0.0426	045 ± 0.14	*0.0153	0.27 ± 0.14	n.s.
SOCS6	1.75 ± 0.23	*0.0336	0.81 ± 0.23	n.s.	0.86 ± 0.23	*0.0123
STING/TMEM173	2.85 ± 0.11	**** < 0.0001	0.87 ± 0.11	n.s.	0.75 ± 0.11	**** < 0.0001
TNF RII/TNFRSF1B	9.11 ± 0.70	**** < 0.0001	1.28 ± 0.70	n.s.	1.70 ± 0.70	**** < 0.0001
TNFRSF16/NGFR	2.07 ± 0.35	*0.0466	1.55 ± 0.35	n.s.	1.12 ± 0.35	n.s.
TRAIL RII/DR5	3.46 ± 0.65	*0.0131	3.22 ± 0.65	*0.0246	3.316 ± 0.65	n.s.

## Discussion

Mu-class GSTs are the most abundant GST isoforms in *F*. *hepatica*^14^ and their importance as potential vaccine candidates or in the host-parasite relationship has been highlighted in previous studies^15,28,29^. Mu-class GSTs have established roles in phase II detoxification of xenobiotic and endogenously derived toxins in *F*. *hepatica* within the hostile bile environment^30^. Their role in detoxification process is advocated since GSTs constitute as much as 4% of the total soluble protein of adult *F*. *hepatica* with a widespread distribution in the parasite’s tissues mainly in parenchyma^11^. Unlike Mu-class GSTs, the recently described Sigma-class GSTs^11^ are responsible for the production of prostaglandins in mammals and parasitic worms^31,32^. Mu- and Sigma-GST isoforms of *F*. *hepatica* also differ in the manner in which they interact with murine dendritic cells (DCs). Whereas the recombinant sigma-GST induced partial activation of DCs with significant IL6, IL12p40 and MIP-2 secretion, the recombinant Mu-GST variant failed to induce cytokine secretion or show any indication of DC activation^15^. The present study demonstrates that nFhGSTs (Mu) failure in inducing pro-inflammatory cytokines/chemokines is in fact a relevant anti-inflammatory mechanism.

In initial experiments, we examined the effect of nFhGST on the capacity of macrophages to produce pro-inflammatory cytokines in response to LPS-stimulation *in vitro*. Macrophages exert key functions during the immune response, functions for which macrophages must be activated^33^. Thus, macrophages are able to secrete tumor necrosis factor-α (TNFα), interleukin-1β (IL-1β) and other inflammatory mediators, which are required for killing microbes. Hence, in the presence of LPS, the potent endotoxin of Gram-negative bacteria, macrophages do not play a positive role but a deleterious effect. Our observation that murine bone marrow derived macrophages, in the presence of nFhGST, are unable to express TNFα and IL-1β in response to LPS-stimuli clearly indicates that nFhGST exerts a negative impact on the activation of these cells. The suppressive effect produced by nFhGST *in vitro* was further confirmed *in vivo* using two mouse strains from different genetic backgrounds that were challenged with LPS. As expected, animals exposed to lethal LPS doses developed the typical systemic inflammation and succumbed approximately 12 h after the LPS-challenge injection. In contrast, those treated with a single dose of 200 μg nFhGST had enough to prevent the pro-inflammatory cytokine/chemokine storm and significantly prolong the survival rate in the animals.

Our proteomic analysis revealed that nFhGST is a highly purified protein comprised of ~96.17% Mu-GST isoforms. However, the resting ~3.83% in the nFhGST composition comprise cathepsins, thioredoxin peroxidase (TxP) and fatty acid binding proteins (FABP), which have been also recognized as immunomodulatory molecules^9,15,34^. Studies performed with native or recombinant TxP or peroxiredoxin have demonstrated that repeated i.p. injections (6 to 9 times) with 4–5 μg per mouse with each of these proteins are required to induce alternative activation of macrophages *in vivo*^9,34^. Moreover, in our own study with native FABP 15 μg of protein was required to fully suppress the pro-inflammatory responses in C57BL/6 mice exposed to sub-lethal dose of LPS^20^. The single injection of 200 μg nFhGST applied in the present study would represent ~7.6 μg of all these contaminant proteins together. This amount would result be insufficient to significantly provoke noticeable effects on the pro-inflammatory response induced by such high LPS-doses. Thus, although a synergistic effect of these minor contaminants should not be ruled out, it is obvious that the Mu-GST isoforms would be the main protagonist of the powerful suppressive effect observed here against the high concentration of LPS-used to induce the lethal sepsis in the animals. A similar reasoning could be applied to explain the lack of a noticeable activating effect mediated by the Sigma-GST isoform, which also was identified within nFhGST. Sigma-GST, has shown to partially activate dendritic cells inducing IL6 and IL12p40 production and activating NF-κB, as reported by other authors^15^. The lack of noticeable levels of cytokine production in our *in vivo* study and the failure in inducing NF-κB *in vitro* suggests that the presence of Mu-class isoforms could be masking the potential action of GST-Sigma isoform, most likely due to the fact that they are at significantly higher concentration in the protein mixture.

Given that *F*. *hepatica* infection has been reported to suppress Th1 responses in concurrent bacterial infections demonstrating its anti-inflammatory effect *in vivo*^35,36^, our findings propose that nFhGST could play relevant roles in parasite immunomodulation. Thus, nFhGST could be one of the antigens that *F*. *hepatica* employs to suppress the Th1-arm of the immune response, which may benefit the parasite survival within the host. This is consistent with the observation that native *F*. *hepatica* GSTs have been directly implicated in the decrease of the proliferative response of mononuclear cells in response to convanavalin-A (ConA) and the inhibition of nitric oxide production by murine peritoneal macrophages^12^. However, we acknowledge that, although in the context of sepsis, the suppression of inflammatory response mediated by *F*. *hepatica* antigens could be advantageous; this phenomenon carries serious implications for the control of other concurrent infections with *F*. *hepatica*. Studies have demonstrated that this parasite suppresses IFNγ altering the response to tuberculosis^10,37^ and delaying bacterial clearance in bystander infection with *Bordetella pertussis*^35,36^.

The Mu-class GST have established roles in general phase II detoxification of xenobiotic and endogenously derived toxins in *F*. *hepatica* within the host bile environment a role that clearly dependent of their enzymatic activity^38^. For this reason, the observation that denaturation by boiling only slightly abrogates the capacity to block TLR4 was unexpected. In the study performed by Dowling *et al*.^15^ researchers tested the ability of a conformationally intact proteolytically inactive variant of cathepsin-L1 (rvFhCL1) in DCs and found that, in contrast to the active molecule, the inactive one failed to alter the expression levels of a number of surface cell markers. To our knowledge, this is the first time that a helminth enzyme retains its capacity to block the NF-κB activation via multiple TLRs after being subjected to extreme temperature conditions. This finding is of great importance and suggests that the anti-inflammatory property of FhGST is due to lineal protein sequence or carbohydrates. If in the near future nFhGST, or any derivate of this molecule, is formulated as a therapeutic anti-inflammatory option, data showed herein concerning thermal stability of the nFhGST function could be important for the development of scaling up protocols, transportation and storage management. Moreover, the finding that nFhGST exerts its suppressive effect via multiple TLRs and that such suppressive effect is observed when cells are treated with nFhGST up to 3 hours after the stimulation with HKLM, LPS or CL075 suggests that this protein exhibits a broad spectrum of action and could eventually, not only be used as prophylactic drug to prevent the development of inflammatory responses, but also as a therapeutic drug to suppress the inflammation mediators at early phases of the bacterial insult.

In an attempt to elucidate whether nFhGST exerts its anti-inflammatory effect by binding to the LPS-endotoxin, we developed an inhibition ELISA assay that pretended to block the specific binding between endotoxin and specific IgG-antibodies raised against the carbohydrate core of the endotoxin using nFhGST. If nFhGST were binding to the endotoxin, it would have saturated the binding sites on the endotoxins coating the microwell, making them inaccessible to the antibody and consequently the absorbance values would be reduced proportionally to the amount of protein added. In the past we have used a similar approach to demonstrate the identity of two molecules and demonstrate that two molecules are able to bind to the same analyte^18,39^. The fact that nFhGST was unable to reduce the absorbance value in this assay (even by a small amount) clearly demonstrates that nFhGST does not bind to LPS. Thus, nFhGST would then have to necessarily bind to the LPS-receptor. However, given that in the activation of TLR4 by LPS various co-receptors (CD14 and MD2)^40^ participate, nFhGST could indistinctly be binding to any of these molecules, which would result in the blockage of the entire TLR4-signaling cascade. Moreover, given the observed effect against a number of TLR-ligands, it might mean that nFhGST binds to multiple receptors preventing that ligands interact with cell. Other helminth molecules, for example the antigen known as ES-62, which is a protein secreted by the filarial nematode *Acanthocheilonema vitae*, also have the ability to simultaneously interfere with various TLRs, since this antigen has shown to inhibit IL-12 production induced by bacterial lipopeptide (TLR2) and CpG (TLR9) independent of the TLR4 pathway^41^. Although this is the first time that *F*. *hepatica* GSTs are tested in terms of the capacity to target multiple TLRs, the observation that nFhGST can suppress TLR2, TLR4 or TLR6 was not surprising because these receptors are expressed on the plasma membrane of immune cells^42^, and thereby they are accessible to be targeted by nFhGST. However, the finding that nFhGST suppressed the activation induced by CL075 was unexpected. CL075 is a synthetic ligand that specifically activates TLR8, which is an endosomal receptor that is typically activated by viral ssRNA^43,44^ or bacterial nucleic acids^45,46^. It remains unclear now how nFhGST targets TLR8 or any other endosomal TLR, but we speculate that it could be transported into the cytoplasm by endocytosis, as has been reported occurs for choleric toxin and other molecules of similar molecular weight^47^. Alternatively, nFhGST could be targeting proteins participating in the NF-κB signaling cascade that are common to multiple TLR-pathways, as it was further demonstrated in our subsequent proteomic array.

Given we had demonstrated that nFhGST significantly suppressed the NF-κB activation induced by whole *K*. *pneumoniae* bacteria via multiple TLR-engagement, we proceeded to identify proteins from the NF-κB pathway that could be suppressed by nFhGST using a fast, simple and sensitive proteomic tool that simultaneously detected the relative levels of a large number of proteins involved in the NF-κB signal transduction. This strategy leads us to better understand the underlying mechanism of nFhGST’s suppressive nature. Results demonstrated that nFhGST significantly downregulated the total cell content of various proteins in the canonic NF-κB pathway, including TLR2 and the adapter molecule MyD88, which have shown to be crucial in pathological process caused by sepsis^48^ and autoimmune diseases^49^. The suppression of TLR2 and MyD88 by nFhGST is consistent with the observation that helminth products modulate TLRs favoring the development of the immune responses by TLR2, TLR4 and MyD88 independent mechanisms^50,51^. Moreover, the observation that nFhGST also suppresses the *K*. *pneumoniae*-induced expression of RelA (p65), cRel, NEMO, JNK2, NF-κkB1 (p105), NF-κB2 (p100), RelB and p52, as well as also members of the IκB protein family that are responsible for regulating the activity of NF-κB indicates that it exerts a redundant suppressive effect on multiple proteins of both canonic and non-canonical NF-κB pathways.

nFhGST also suppressed *K*. *pneumoniae*-induced NF-κB activation by downregulating the total cell content of proteins that participate in JAK/STAT pathway. STAT1 is one of the most important players in the IFNγ signaling important for the classic activation of macrophages, which is typical in bacterial infections but not in helminth infections. Although there are no studies exploring the role of STAT1/STAT2 during the polarized Th2-immune responses elicited during an active *F*. *hepatica* infection, in schistosomiasis it is well known that STAT1 is involved in the Th1 response that is prompted during the earliest phase of infection, a response that is initiated by soluble egg antigens (SEA) derived by laying eggs in the lung and liver, which makes our finding interesting since it does not follow other helminth patterns of action.

Importantly, tumor necrosis factor receptor-II (TNFRII) and the tumor suppressor p53 (pS46) are other proteins downregulated by nFhGST, which are strongly associated with the high incidence of death by sepsis. Studies demonstrate that p53 is highly up regulated during sepsis and that the blockage of this protein could prevent cell death during the development of sepsis/septic shock^52^. Therefore, our finding suggesting that nFhGST suppressed the levels of TNFRII and p53 (pS46) simultaneously is relevant and could have therapeutic implications.

In summary, this study is the first to report that native *F*. *hepatica* GST proteins comprised mainly of Mu-class isoforms are powerful anti-inflammatory molecules. nFhGST is able to suppress the LPS-induced cytokine/chemokine storm and significantly increase the survival rate in a mouse model of septic shock. Importantly, nFhGST achieves its potent effect, not only targeting TLR4, but also multiple TLRs and effector molecules involved in the NF-κB signaling pathway. We confirmed the anti-inflammatory effect of this protein has a broader spectrum of action and this effect was found to be independent of its enzyme activity or secondary structure integrity, which might suggest that ΔFhGST should be as effective as nFhGST preventing the septic shock in animals exposed to LPS or whole bacteria. Experiments to confirm this assumption are being scheduled. The demonstration that nFhGST suppresses the NF-κB pathway by targeting multiple TLRs pathways with the capacity to modulate innate and adaptive immune responses supports the view that helminth antigens have developed multiple strategies to control the outcome of infections. Since Mu-GST proteins are universally expressed by all helminth parasites, this suggests the existence of common immunomodulation mechanisms. More studies are required to fully understand the immunological implications of using Mu-GST proteins of *F*. *hepatica* as target drug to control sepsis or any other inflammation-based disorder. Understanding how this antigen affects the maturation and function of macrophages and other immune cells may shed light on immune modulatory mechanisms collectively associated with *F*. *hepatica* infection and its implication during co-infections.

## Methods

### Ethics Statement

All the animal studies were performed at the Animal Resources Center of the Medical Sciences Campus, University of Puerto Rico in accordance with guidelines and protocols approved by the Ethics Institutional Animal Care and Use Committee (MSC-IACUC, Protocol No. 7870215). Female C57BL/6 and BALB/c mice of 6–8 weeks of age were obtained from Charles River Laboratories. All procedures applied to animals (bleedings, injections and euthanasia) were performed under deep anesthesia using a cocktail of 100 mg/kg body wt. of ketamine + 10 mg/kg body wt. of xylaxine given intraperitoneally. Depth anesthesia was verified by lack of withdrawal reflex.

### Whole bacteria preparation

Whole heat-killed bacteria were prepared from normal and multidrug resistant bacterial strains *Enterococcus faecalis* and *Klebsiella pneumoniae* isolated from a local clinical laboratory. Bacteria were cultured in Luria Bertani (LB) broth for approximately 8 hours and then were killed by boiling for 15 min in a water bath.

### Purification of native *F*. *hepatica* glutathione *S*-transferase (nFhGST)

*F*. *hepatica* GST was purified from a crude soluble extract of adult fluke using 5/5 HP GSTrap column (GE Healthcare) as previously described^16^. The CDNB (1-chloro-2, 4-dinitrobenzene) enzymatic activity assay was used for monitoring the binding efficiency and recovery of the protein during the purification process. Purified nFhGST was desalted against PBS using a PD-10 column (Sephadex-G25, GE Healthcare) and then concentrated by AMICON ultrafiltration system using an YM-10 membrane (cut-off > 10 kDa). An aliquot of purified nFhGST was subjected to 15% SDS-PAGE and protein composition was visualized by Coomassie Blue staining. Protein purity and identification of possible GST-isoforms was performed by nano-LC-MS/MS on a Q Exactive equipped with an Easyn-LC 1000 HPLC System (Thermo Scientific), USA). Endotoxins were removed from nFhGST batches using GenScript ToxinEraser™ polymyxin-B (PMB) resin according to the manufacturer’s instructions and the absence of endotoxin was confirmed using the Chromogenic *Limulus* Amebocyte Lysate QCL-1000 Assay (Lonza, Walkersville, MD). nFhGST was then concentrated by AMICON Ultra-15 Centrifugal filter unit and adjusted to 1 mg/ml as determined using the Pierce protein bicinchoninic acid (BCA) method (Pierce, Cambridge, NJ). Purified endotoxin-free nFhGST was stored in aliquots at −20 °C until use.

### Anti-nFhGST serum production

C57BL/6 mice (n = 4) received 3 subcutaneous injections with 20 μg nFhGST mixed with an equal amount of water-in-oil emulsion Montanide ISA51 adjuvant (Seppic Inc, Fairfield, NJ). The injections were applied two weeks apart. Animals were bled out one week after the last injection and the sera from each individual animal were pooled. Anti-serum had antibody titers of ~1: 25,600 when titrated by ELISA against nFhGST.

### Thermal stability and circular dichroism measurements

Fifteen micrograms of nFhGST in 50 mM phosphate buffer, pH 8.0 were heated for 10 min at 95 °C in a water-bath. Circular dichroism (CD) measurements in the far-UV region (190–350 nm) were performed with a Jasco J-1500 CD spectrometer and protein concentrations of 0.1 mg mL^−1^ in 50 mM phosphate buffer, pH 8.0 in the 190–250 nm ranges at 20 °C and 95 °C using 0.1 cm pathlength cell.

### Mouse model of endotoxemia

C57BL6 and BALB/c mice were allotted into groups of 12 animals each. The day of procedure animals were anesthetized and a blood sample of 0.2 ml was taken from the orbital sinus of each animal using a capillary microtube to establish the base-values of all cytokines measured in serum. Following bleeding, animals received a single intraperitoneal (i.p.) injection with 200 μg nFhGST one hour before a lethal i.p. injection with 60 mg/Kg body wt^24^. or 7 mg/Kg of body wt. of LPS (*E*. *coli* 0111:B4), respectively^25^. Control groups received injection with PBS, nFhGST or LPS only (i.p.). Following initial administration of experimental treatments animals were placed in a cage over a heating pad and observed until full recovery. Following recovery animals were closely monitored for mortality during 48 h. Monitoring was performed every hour for the following 12 h after the LPS-challenge and then every 4 h until the end of the experiment (48 h after the LPS-challenge injection) at this time point all animals were bleed by terminal cardiac puncture and euthanized by cervical dislocation under deep anesthesia. Animals that during the monitoring process showed signs such as inability to maintain upright position, physical inability to obtain food and/or water, anorexia and or clinical dehydration, agonal breathing and cyanosis or unconsciousness with no response to external stimuli were immediately anesthetized for bleeding followed by euthanasia as described above.

### Cytokine/Chemokines Measurements

Serum samples collected from mice in the endotoxemia *in vivo* model were tested in triplicate with a multiplex solid-based immunoassay (Cytokine 20-Plex mouse panel, Invitrogen, Carlsbad, CA) using the Luminex MAGPIX® system. Data was analyzed with the Bio-Plex Manager 6.1 software (BIO-RAD, Hercules, CA) using a 5-parameter logistic curve.

### Screening of TLR-activation using THP1-Blue-CD14 transfected cells

To investigate the effect of nFhGST or denatured FhGST (ΔFhGST) on the activation of multiple TLR-pathways, we used a human monocyte cell line (THP1-Blue-CD14), which stably expresses the human CD14 gene, as well as encodes for a macrophage specific differentiation antigen and multiple TLRs including TLR2, TLR4, TLR6 and TLR8. Cells are transfected with a reporter gene, secreted embryonic alkaline phosphatase (SEAP) driven by the NF-κB promoter (Invivogen, San Diego, CA). Cells were seeded in 96-well flat-bottom plates at 1 × 10^6^ cells/well in 100 μl of RPMI supplemented with 10% heat-inactivated fetal bovine serum fetal bovine serum (FBS), 50U/ml penicillin, 50 μg/ml streptomycin, and 2mM L-glutamine. In the stimulation experiments cells were treated with nFhGST (0.5 to 20 μg/ml) or whole *K*. *pneumoniae* (1.22 × 10^8^ cells/ml) or *E*. *faecalis* (1.14 × 10^9^ cells/ml) and incubated at 37 °C, 5% CO_2_ for 18 h. Cells treated with TLR-agonist at the concentration recommended by the manufacturer (Invivogen, USA) were also used as activation control. The TLR-agonists used in the study were lipopolysaccharide (LPS) (1 μg/ml), heat-killed *L*. *monocytogenes* (HKLM) (10^8^ cells/ml), synthetic diacylated lipoprotein (FSL-1) (1 μg/ml) and orthiazoloquinoline (CL075) (10 μg/ml). In the inhibition experiments, cells were cultured with different concentrations of nFhGST or ΔFhGST for 30 min and then stimulated with HKLM, LPS, FSL-1, CL075 or whole bacteria. Cells treated with PBS were used as negative control and cells incubated with polymyxin-B (PMB) (100 μM) or Chloroquine (100 μM) was used as antagonist controls. In other experiments, cells were first stimulated with LPS (1 μg/ml) for 30 min, 1 h and 3 h prior to the addition of nFhGST (10 μg) and incubated 18 h. To quantify the levels of stimulation or inhibition, 20 μl of medium of each well was transferred to a clean 96-well flat-bottom plate, the QB reagent was added (180 μl/well) and the incubation prolonged for 5 h. Absorbance readings were done at 655 nm (A_655_). The inhibition percentage in the levels of SEAP secreted to culture media were calculated by the formula R (%) = 1 − [(A − C)/(B − C)] × 100, where A is the mean A_655_ of three replicates obtained when cells were cultured with nFhGST, and B is the mean A_655_ value obtained when cells were exposed to TLR-ligands, and C is the mean A_655_ of three replicates obtained when cells were stimulated with PBS.

### TLR4 stimulation in the presence of anti-nFhGST antibody

To ascertain whether the presence of anti-nFhGST antibodies can neutralize the effect of nFhGST, THP1-XBlue CD14 cells were cultured with the anti-nFhGST sera (endotoxin levels <0.1 EU/ml) obtained by SC or IP injections at dilutions ranging from 1:25 to 1:20 in endotoxin-free PBS and 30 min later were incubated with nFhGST (10 μg) followed by the stimulation with LPS (1 μg/ml) another 30 min later. Cells were then incubated at 37 °C, 5% CO_2_ for 18 h, then incubated with the QB-reagent for 5 h and the absorbance read at 655 nm as described above. For controls, cells were cultured only with the anti-nFhGST serum and stimulated with LPS or only with the anti-serum, PBS or nFhGST.

### Isolation and treatment of mouse bone marrow–derived macrophages

BMDM were collected from femoral and tibial shafts of mice by flushing with 3 ml cold sterile PBS. The cell suspensions were passed through a sieve to remove large clumps, washed three times with sterile complete DMEM (supplemented with 20 mM L-glutamine, 1 ml penicillin and streptomycin/100 ml medium, and 10% heat-inactivated FCS; Sigma-Aldrich). Cells were adjusted to 1 × 10^6^ cells/well with differentiation medium (complete RPMI 1640 supplemented with 20 ng/ml M-CSF; R&D Systems) and cultured in 24-well plates (Nunc) at 37 °C, 5% CO_2_. On day 3 of culture, non-adherent cells were removed and the adherent cells were placed in fresh differentiation medium, and the incubation was prolonged for 7 days to cause full maturation of macrophages, which was assessed by FACS analysis and F4/80 surface Ag expression. BMDM were seeded into 24-well plates (Nunc) at 1 × 10^6^ cells/ml in complete RPMI and then treated with 10 μg FhGST for 30 min before being exposed to LPS (100 ng/ml). Control cells were treated with PBS, FhGST, or LPS alone.

### Quantitative real-time RT-PCR (qPCR)

Total RNA was extracted using a RNA isolation kit (Qiagen) kit, followed by treatment with Turbo DNA free endonuclease (Ambion, Grand Island, NY) to remove contaminating genomic DNA. RNA was quantified using a Nanodrop-1000 spectrophotometer (Thermo-Scientific, USA) and a high-capacity RNA-to-cDNA kit (Applied Biosystems, Carlsbad, CA) to perform the reverse transcription. cDNA was amplified using a StepOne Plus Real-Time PCR system (Applied Biosystems). cDNA was equivalent to 5 ng total RNA and SYBR green PCR master Mix (Applied Biosystems). The cycling conditions were as follows: 95 °C for 15 min followed by 40 cycles of 95 °C for 15 s, 55 °C for 30 s, and 72 °C for 30 s. The primers used for each gene are listed in Table 2. Primer concentration was optimized and dissociation curves were generated for each primer set to verify the amplification of a single PCR product. qPCR experiments were conducted in triplicate using a StepOne Plus real-time PCR system (Applied Biosystems). The 2^−ΔΔCt^ method^53^ was used to quantify relative gene expression using GAPDH as an internal control and expressed as fold change relative to expression in the cells stimulated with PBS. The values reported are the mean of three replicates. The SD of the mean is shown as error bars in each group.

**Table 2:** Primers used for cytokines analysis using real-time RT-PCR.

Primer	Sequence 5′ to 3′
ACTB	Sense: 5′-AGAAAATCTGGCACCACACC-3′
	Antisense: 5′-GGGGTGTTGAAGGTCTCAAA-3′
IL-1β	Sense: 5′-GCTCGCCAGTGAAATGATGG-3′
	Antisense: 5′-GTCCTGGAAGGAGCACTTCAT-3′
TNF-α	Sense: 5′-TGGGATCATTGCCCTGTTGAG-3′
	Antisense: 3′-TCTAAGCTTGGGTTCCGACC-3′

### Capacity of nFhGST to bind an endotoxin carbohydrate core

To investigate whether nFhGST could bind to the endotoxin we used an EndoCab^R^ IgG assay (HK504-IgG, Hycult Biotech). This assay, which is designated for quantitative determination of endotoxin-core antibodies in serum or plasma, was transformed into an inhibition endotoxin core antibody assay as follows: 100 μl of several nFhGST concentrations (2.5 μg to 20 μg) prepared in the supplied dilution buffer was added in duplicate to microtiter wells coated with equimolar amounts of endotoxin rough-lipopolysaccharides from four Gram negative bacterial species. After 1 h incubation at 37 °C, unbound protein to the coated-endotoxin was removed by suction and the micro wells were washed three times with the provided washing solution. Further, the IgG antibody provided as standard, which specifically binds to the endotoxin-core coated to the wells, was added (100 μl/well) diluted 1:8 followed by another incubation of 1 h at 37 °C. After another washing step, the peroxidase conjugate antibody provided in the kit was added and incubated at 37 °C for 1 h. Wells were again washed three times prior the addition of the TMB substrate solution. After 30 min of incubation at room temperature in the dark the reaction was stopped with stop solution and absorbance at 450 nm was read. Wells incubated with the IgG standards in absence of nFhGST were used as positive controls. Wells incubated only with nFhGST in absence of standards or wells incubated with wash buffer in absence of nFhGST and standards were used as negative controls.

### Effect of nFhGST on the NF-κB pathway

To investigate the possible impact of nFhGST on the proteins participating in the NF-κB signaling pathway, THP1-Blue CD14 cells (1 × 10^6^ cells/well) were treated in triplicate with PBS, nFhGST, *K*. *pneumoniae*, or the mixture nFhGST + *K*. *pneumoniae* for 18 h at 37 °C, 5%CO_2_ as described above. After incubation, cells were harvested and lysed in the presence of Halt^TM^ Protease and Phosphatases Inhibitor Cocktail (Thermo Scientific, USA) and immediately analyzed in a proteome profiler array designed for studying the human NF-κB pathway (R&D Systems, Inc., USA). Approximately 250 μg of each sample were incubated overnight onto a dot blot membrane of the human NF-κB pathway array. The array consists of capture and control antibodies specific for 45 proteins that have been spotted in duplicate on nitrocellulose membranes (Fig. S1). Cell lysates were diluted and incubated overnight with the membrane array. The membrane was washed to remove unbound proteins followed by the incubation with a cocktail of biotinylated detection antibodies. Next, streptavidin-HRP and chemiluminescent detection reagents were applied, and a signal was produced at each capture spot corresponding to the amount of protein bound. The dot blot images were acquired simultaneously in a Chemidoc MP Imaging system (Bio Rad, Hercules, CA) and densitometry analysis was performed on all the arrays using ImageJ software (https://imagej.nih.gov/ij/download.html). Values were normalized to the PBS-treated control and all values were expressed in arbitrary units over the PBS-treated cells.

### Cell viability

To determine whether the optimized nFhGST concentration affects cell viability, BMDMs and THP1-Blue CD14 cells were seeded at 1 × 10^6^ cells/well in 96-well flat bottom plates and treated with LPS (1 μg/ml), nFhGST (10 μg/ml) or nFhGST (10 μg/ml) + LPS (1 μg/ml) for 24 h at 37 °C. Following incubation, cell viability was assessed by adding 50 μl XTT (sodium 3′-[1-(phenylaminocarbonyl)-3,4 tetrazolium]-bis(4-methoxy-6-nitro) benzene sulfonic acid hydrate) labeling reagent (Roche Life Science, USA) to each well. The absorbance was read at 480 nm.

### Statistical analysis

Survival was analyzed by Kaplan-Meier analysis. All data were analyzed for normality prior to statistical testing. For comparisons between multiple groups we used ANOVA and for comparison between two groups, the Student’s *t*-test was used. All statistics were done with GraphPad Prism 6.0 (San Diego, CA).

### Methods Statement

Authors declare that all experiments and procedures performed with animals were performed in accordance with relevant guidelines and regulations of the Institutional Animal Care and Use Committee of University of Puerto Rico, Medical Sciences Campus and approved by this committee (Protocol Number: 7870215).

## Supplementary information

Supplementary Figures
