# Supplementary material for: Fasciola hepatica GST downregulates NF-κB pathway effectors and inflammatory cytokines while promoting survival in a mouse septic shock model
Source: Sci Rep. 2019 Feb 19;9:2275. doi: 10.1038/s41598-018-37652-x (PMC6381083; doi:10.1038/s41598-018-37652-x)
Supplement: Supplementary file 1 — Supplementary Figures [file 41598_2018_37652_MOESM1_ESM.docx]

**SUPPLEMENTARY FIGURES AND TABLE**

***Fasciola hepatica* GST down regulates NF-κB pathway effectors and inflammatory cytokines while promoting survival in a mouse septic shock model**

Vasti Aguayo, Bianca N. Valdés Fernández, Madeline Rodríguez-Valentín, Caleb Ruiz-Jiménez, Marcos J. Ramos-Benítez, Loyda B. Mendez and Ana M. Espino
